# Distinct Transcriptomic and Tumor Microenvironment Profiles in Sinonasal Mucosal Melanoma and Aggressive Cutaneous Melanomas

**DOI:** 10.3390/cancers16244172

**Published:** 2024-12-14

**Authors:** Manuel Molina-García, María Jesús Rojas-Lechuga, Teresa Torres Moral, Jaume Bagué, Judit Mateu, Cristóbal Langdon, Joan Lop, Vinícius Gonçalves de Souza, Llúcia Alós, Mauricio López-Chacón, Sebastian Podlipnik, Cristina Carrera, Josep Malvehy, Isam Alobid, Rui Milton Patricio da Silva-Júnior, Susana Puig

**Affiliations:** 1 Institut d’Investigacions Biomèdiques August Pi i Sunyer (IDIBAPS), 08036 Barcelona, Spain; manmolina@recerca.clinic.cat (M.M.-G.); patricioda@recerca.clinic.cat (R.M.P.d.S.-J.); 2 University of Barcelona (UB), 08036 Barcelona, Spain; mrojas@clinic.cat; 3 Melanoma Unit, Dermatology Department, Hospital Clinic of Barcelona, 170 Villarroel, 08036 Barcelona, Spain; 4 Otorhinolaryngology Department, Hospital Clínic de Barcelona, CIBERES, IDIBAPS, Universitat de Barcelona, 08036 Barcelona, Spain; 5 Centre of Biomedical Research on Rare Diseases (CIBERER), Instituto de Salud Carlos III, 28029 Barcelona, Spain; 6 Otorhinolaryngology Department, Hospital Sant Joan de Déu, 08950 Barcelona, Spain; 7 Pathology Department, Hospital Clínic de Barcelona, IDIBAPS, 08036 Barcelona, Spain; 8 Molecular Oncology Research Center, Barretos Cancer Hospital, Barretos 14784-400, Brazil

**Keywords:** sinonasal mucosal melanoma, cutaneous melanoma, transcriptomic profiles, prognostic markers, gene expression, tumor microenvironment

## Abstract

**Background/Objectives**: Sinonasal mucosal melanoma (SNMM) is a rare and aggressive melanoma subtype with a notably poor prognosis compared to cutaneous melanoma (CM). Despite advances in molecular characterization, SNMM remains underexplored, posing a clinical challenge and highlighting the need for detailed molecular profiling. This study aimed to identify the molecular features of SNMM, elucidate its clinical behavior and prognostic implications, and provide insights for improved therapeutic strategies. **Methods**: This retrospective study analyzed 37 primary melanoma tumors diagnosed at the Hospital Clinic of Barcelona. Gene expression was examined using 1402 immuno-oncology-related probes through next-generation sequencing. Hierarchical clustering analysis (HCA), differentially expressed genes (DEGs), gene set enrichment analysis (GSEA), and the xCell algorithm were performed. The statistical methods comprised descriptive statistics, clinical variable associations, and survival analyses. **Results**: HCA revealed two primary clusters. Cluster A exclusively contained CM tumors (20/24), while cluster B included all SNMMs (13/13) and some CMs (4/24). Cluster B showed a higher average age at diagnosis (*p* = 0.018), higher mitotic index (*p* = 0.0478), fewer *BRAF* mutations (*p* = 0.0017), and poorer melanoma-specific survival (*p* = 0.0029). Cluster B showed 602 DEGs with cell cycle pathways enriched, immune pathways diminished, lower immune scores (*p* < 0.0001), and higher stromal scores (*p* = 0.0074). **Conclusions**: This study revealed distinct molecular characteristics and an altered tumor microenvironment in SNMMs and certain aggressive CMs. Identifying specific genes and pathways involved in cell cycle progression and immune evasion suggests potential prognostic markers, offering new avenues for enhancing treatment strategies and improving patient survival rates.

## Simple Summary

Melanoma is a cancer that originates from melanocytes, the cells responsible for producing the pigment melanin. Sinonasal mucosal melanoma (SNMM) is a rare and aggressive form of melanoma occurring in the nasal cavity. Unlike the more common cutaneous melanoma (CM), which affects the skin, SNMM is less understood and has a poorer prognosis. This study aimed to explore the genetic characteristics of SNMM to better understand why this cancer behaves so aggressively and to identify potential ways to improve treatment. By analyzing tumors from patients, we found that SNMM has a unique genetic profile, with increased activity in genes related to cell growth and reduced activity in immune-related genes. This suggests that SNMM could evade the immune system, which may explain its poor prognosis. Our research provides valuable insights into SNMM and may help develop new treatments to improve the survival chances of patients with this challenging cancer.

## 1. Introduction

Sinonasal mucosal melanoma (SNMM) represents a rare subtype of melanoma, distinct in its clinical presentation, prognosis, and molecular characteristics from the more common cutaneous melanoma (CM) [1]. Despite advancements in melanoma treatment, SNMM continues to pose a significant clinical challenge due to its aggressive nature, poor survival outcomes and limited systemic therapy options [2].

The aggressiveness of SNMM is reflected in its markedly lower patient survival rates [3]. The recent literature indicates a 5-year melanoma-specific survival (MSS) rate below 40% [4]. This is significantly lower than the survival rates observed in CM (91.2%), demonstrating the need for a focused analysis of SNMM [5]. The high recurrence rates and metastatic potential further compound the challenge, with our local series showing a locoregional recurrence rate of 46% and a metastasis rate of 36% within a median follow-up period of 39.6 months [6].

SNMM often presents at an advanced stage and is diagnosed based on histological features and immunohistochemistry, showing a high expression of melanocytic markers, such as SOX10, which is present in 99% of cases [7]. Immunohistochemical studies have shown that all SNMMs express at least one melanocytic marker, such as HMB-45, MELAN A, S100 protein, SOX10, or tyrosinase [8]. Key molecular markers include preferentially expressed antigen in melanoma (PRAME) [9], c-Myc, and KIT (CD117) [10].

Several clinical–pathological factors are associated with the prognosis of SNMM, including TNM (tumor, node, metastasis) staging, treatment modalities, surgical margins, mitotic index, and specific gene mutations. For instance, SNMM exhibits mutations in about 20–30% (*NRAS*) and 0–8% (*BRAF*) of cases [1], which contrasts with the mutation frequencies in CM, where they are observed in approximately 20% and 40% of cases, respectively [11,12], while *KIT* mutations have been reported in 8.5% (95% CI: 8.1–9.0) of SNMMs in a recent systematic review including 24 series and 787 patients [12]. Additionally, SNMM exhibits a lower tumor mutational burden and higher copy number variations (CNVs) [13,14], further highlighting its distinct molecular profile and underscoring the importance of reaching deeper molecular insights into SNMM [5,15].

Despite advances in molecular research, a comprehensive molecular classification of SNMM remains incomplete, especially regarding its molecular features. This study compares the transcripts of an immuno-oncology gene panel in a cohort of SNMM and CM patients, aiming to identify molecular profiles that could shed light on their clinical behavior and prognostic implications. This study reveals that SNMMs and certain aggressive CMs exhibit distinct transcriptomic profiles characterized by upregulated cell cycle-related pathways and downregulated immune system-related pathways. It also demonstrates significant alterations in the tumor microenvironment, with decreased populations of CD4+ and CD8+ T cells and dendritic cells (DCs), alongside an increase in endothelial cells. These findings fit with the aggressive behavior and poorer prognosis observed in both SNMMs and certain aggressive CMs.

## 2. Materials and Methods

### 2.1. Study Design, Patients, and Inclusion Criteria

This retrospective study was conducted at the Hospital Clinic of Barcelona (HCB), adhering to established ethical guidelines, notably the Declaration of Helsinki. The HCB Ethics Committee granted ethical approval (#HCB/2020/1454 and #HCB/2018/1074), and written informed consent was obtained from all participants or their legal guardians. The patient cohort comprised individuals diagnosed at the HCB from April 2010 to February 2021.

The inclusion criteria for SNMM and CM were based on confirmed histopathological diagnoses of melanoma. For SNMM, eligibility was restricted to patients with primary tumors originating within the sinonasal tract. In the case of CM, inclusion was limited to cases classified as superficial spreading or nodular melanoma that required immunotherapy at some point in their evolution.

Clinical, demographic, histopathologic, and disease status data were extracted from medical records. The index date was set at the diagnosis, and the latest follow-up was updated on 2 February 2024 (Supplementary Table S1). Melanoma diagnosis was based on comprehensive clinical evaluation, dermoscopic assessment or nasal endoscopy, and histopathological analysis, adhering to the melanoma AJCC 8th edition guidelines (Mucosal Melanoma of the Head and Neck [16] or Skin Melanoma [16]). The histopathological data included ulceration presence, Breslow index (for CM), mitotic index, tumor *BRAF* mutation status, and other somatic mutations (Supplementary Table S2). Detailed histopathological analyses, including an assessment of mitotic activity, nuclear pleomorphism, necrosis, cytoplasmic abundance, pigmentation, cell morphology, lymphocytic inflammation, and tumor-infiltrating lymphocytes (TILs), were conducted on digitized hematoxylin and eosin (H&E)-stained sections, as described in the Supplementary Materials.

### 2.2. Sample Preparation, Library Construction, Sequencing, and Data Processing

Tumor samples were collected during surgical procedures, and routine histopathological examination and immunohistochemical studies were performed to identify melanocyte markers and markers of proliferation and aggressiveness. Hematoxylin and eosin (H&E) staining was performed to demarcate tumor areas precisely. Formalin-fixed paraffin-embedded (FFPE) blocks of the tumor tissue were sectioned (5 µm thick sections) for molecular analysis. The HTG EdgeSeq was employed on unstained slices, macrodissecting a tumor area of 12–30 mm^2^. Quantification of the mRNA expression from the macrodissected tissue was performed. Sample processing, library construction, and sequencing were performed according to the manufacturer’s protocol. For FFPE mRNA binding, the sample was permeabilized with HTG lysis buffer, adding gene-specific Nuclease Protection Probes (NPP) from the Precision Immuno-Oncology Panel (1402 probes, (HTG, Tucson, AZ, USA)) to form probe–target RNA heteroduplexes. Non-hybridized mRNA and excess NPPs were digested with S1 nuclease, followed by PCR barcoding. Sequencing was performed by the Illumina NextSeq 550 (Illumina, San Diego, CA, USA) sequencer. Data processing was carried out from FASTQ Files obtained from the sequencer. The data were parsed and aligned to the probe list using the HTG Parser (HTG, Tucson, AZ, USA). The quality control metrics included the positive control read counts (QC0), total read counts per case (QC1), and standard deviations among the case probe read counts (QC2).

### 2.3. Unsupervised Analysis

A DESeqDataSet (dds) object was created from the transcript total read counts (RCs) matrix, incorporating the sample metadata. The ensuing process entailed the computation of counts per million (CPM) values, followed by their transformation into log_2_ (CPM) values using the DESeq2 package (v. 1.38.3). The log_2_ (CPM) values were used to calculate the Z-score for each gene. Hierarchical clustering analysis (HCA) was performed with the Ward.D method; the distances were calculated with the Spearman correlation method applied to the Z-score matrix. To assess the robustness and stability of the clusters identified, a bootstrap analysis with N = 10,000 replicates was conducted. For gene HCA, the Ward.D clustering method and Manhattan distances were used. Additionally, the metadata were illustrated using a color code, and a heatmap was generated with the ComplexHeatmap package (v. 2.15.4). All scripts were executed using the R language (v. 4.2.3) in the RStudio environment (v. 2023.06.0+421).

### 2.4. Differential Expression Analysis

Differentially expressed genes (DEGs) were identified among specific conditions using the DESeq2 R package. The RCs, clinical data, and sample HCA information were integrated into metadata to create the DESeqDataSet. A negative binomial distribution-based model (Rlog) was fitted to normalize the sample-specific variability. Subsequently, the DESeq function was applied to perform the specific DEG analysis.

### 2.5. Gene Set Enrichment Analysis (GSEA)

GSEA was conducted on genes identified from the DEG analysis with an adjusted *p*-value (*p*-adj) < 0.05. All probes (1402) were renamed (Gene Symbol) in accordance with the equivalent classification in the HUGO Gene Symbols and Entrez IDs, utilizing the clusterProfiler package. Probes lacking equivalences or not corresponding to unique genes (e.g., those detecting multiple isoforms or gene families) were reannotated or excluded. The complete list of gene nomenclature equivalences and eligibility is available in Supplementary Table S3. A total of 1381 genes with the respective stat values were used as input for GSEA analysis, employing the Gene Ontology (GO) Biological Processes (BP) as the functional database. The analysis was conducted using the ‘gseGO‘ function from the clusterProfiler package (v.4.6.2), including additional parameters such as the gene set size, Benjamini–Hochberg correction (BH), and a statistical significance threshold of *p* < 0.05.

### 2.6. Immune, Stroma, and Tumor Microenvironment (TME) Signatures

Twenty-three HTG EdgeSeq Reveal Immunophenotyping Signatures were applied to Precision Immuno-Oncology Panel data (HTG, Tucson, AZ, USA) to estimate the relative abundance of 19 immune and 4 stroma cell types. All signatures were performed using the xCell algorithm (v. 1.1.0) [17].

### 2.7. Statistical Analysis

Continuous variables were evaluated for normality with the Shapiro–Wilk test; means and standard deviations were reported for the normally distributed data, and medians with interquartile ranges (IQRs) for the non-normal data. The survival time was expressed using the median and IQR. Kaplan–Meier survival analyses were conducted to assess MSS across clusters and subclusters, with differences in the survival rates evaluated using the log-rank test. Categorical variables were summarized as frequencies and percentages and analyzed using the Chi-square (χ^2^) or Fisher’s exact test, as appropriate. The Mantel–Haenszel test for a linear trend was applied in cases of ordinal data. All tests were two-tailed, with an alpha level of 0.05 for statistical significance. Analyses were performed using STATA software v.16.1 (StataCorp, TX, USA) and R language (v. 4.2.3) in the RStudio environment (v. 2023.06.0+421).

## 3. Results

### 3.1. Patients and Clinical Presentation

Our study included 40 patients diagnosed with melanoma; three SNMM cases were excluded from the quality control analysis. Therefore, a final cohort of 37 patients was analyzed (24 CMs and 13 SNMMs). The patients’ ages ranged from 24 to 89 years, with a median age of 63.9 years. The study population was diverse, comprising different ethnicities and both sexes (70.3% male and 29.7% female). The baseline demographic, clinical, dermatological, histopathological, and molecular features of the patients with SNMMs and CMs are summarized in Supplementary Tables S4 and S5.

### 3.2. The Transcriptome Delineates Two Melanoma Profiles Linked to Clinical–Pathological Classification and Prognosis

**Figure 1:**
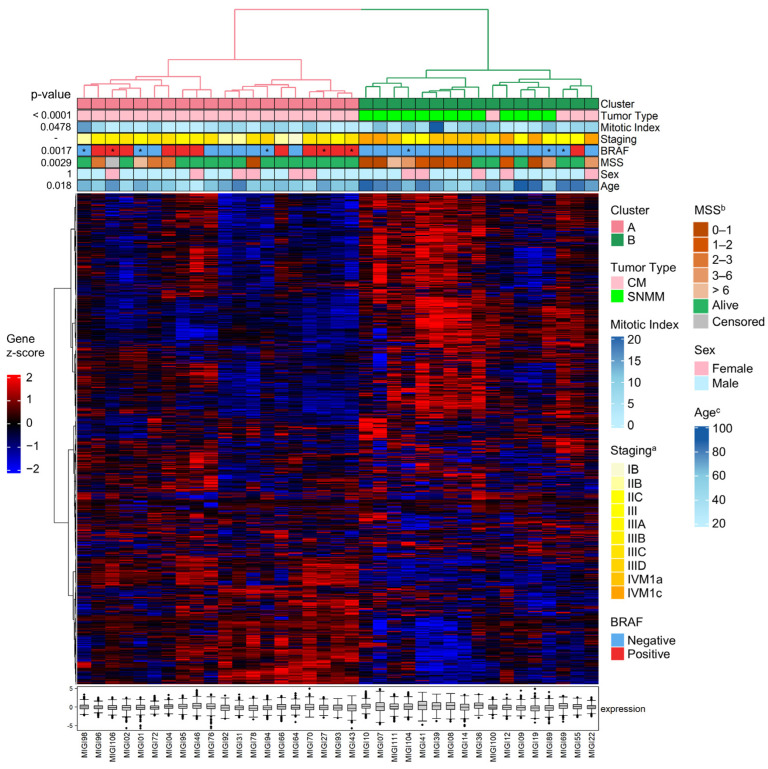
Hierarchical clustering analysis (HCA) of sinonasal mucosal (SNMM) and cutaneous melanoma (CM) tumors. Hierarchical clustering of the samples was performed using Spearman correlation distances and Ward’s method. Gene clustering was based on Manhattan distances and the Ward.D method. Annotations at the top of the heatmap display the clinical–pathological data with a color code. The expression heatmap reflects the gene Z-scores, ranging from low (blue: −2) to high (red: 2) expression levels. The boxplots represent the median expression levels of all genes in the panel for each specific sample, showing the interquartile range and outliers to highlight the overall expression variability within each sample. * The *BRAF* mutation status was obtained from a sample different from the primary tumor. ^a^ Staging at diagnosis; ^b^ melanoma-specific survival (years); ^c^ age at diagnosis.

HCA of the transcriptomic data was conducted using all 1402 probes across 37 tumors. This analysis revealed two tumor clusters with bootstrap support of 70% for each of the two major branches: cluster A (branch #35—exclusively composed of CM tumors) and cluster B (branch #34—enriched for SNMM tumors). Within cluster B, the HCA discerned two branches: one exclusively composed of SNMM tumors (branch #32, subcluster B1) and another mixed, composed of both SNMM and CM tumors (branch #31, subcluster B2) (Figure S1 and Figure 1). The clinical, histopathological, and molecular features of the tumors are illustrated using color coding in Figure 1.

The identified transcriptomic signatures (clusters A and B) were associated with tumor diagnosis (tumor type; *p* < 0.0001), age at diagnosis (*p* = 0.018), mitotic index (*p* = 0.0478), and presence of the *BRAF* mutation (*p* = 0.0017). No significant differences were observed regarding sex. Cluster A was exclusively composed of CM tumors (20/24; 83% CM). Cluster B was a mixed group of SNMM (13/13; 100% SNMM) and CM tumors (4/24; 17% CM) (Figure 1). When subdividing cluster B into subclusters B1 and B2, subcluster B1 was exclusively formed by SNMMs (9/13; 69% SNMM), while subcluster B2 comprised a mix of SNMM (4/13; 31% SNMM) and CM tumors (4/24; 17% CM). The CM tumors in subcluster B2 were predominantly nodular (3/4; 75% CM) with an average mitotic index of 7.8 (SD 2.9), an average Breslow thickness of 6.2 mm (SD 2.9), a mean age at diagnosis of 77.2 years (SD 5.8), and predominantly negative for *BRAF* mutations (3/4).

**Figure 2:**
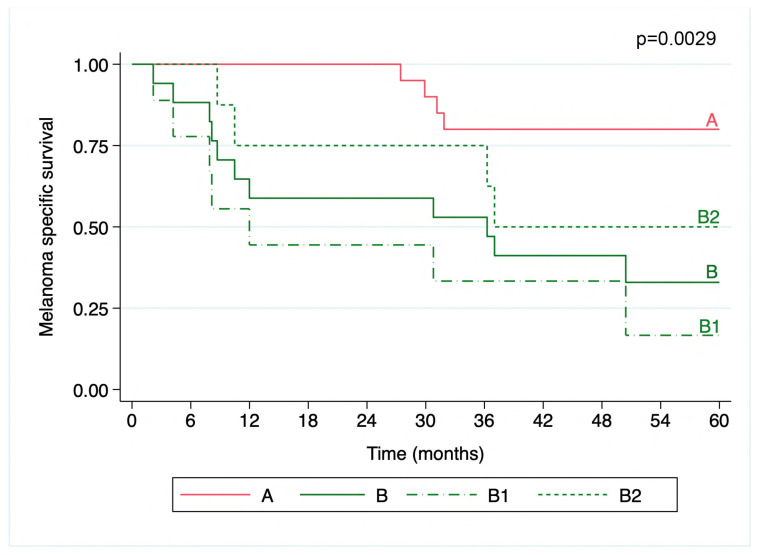
Kaplan–Meier curve of melanoma specific survival over 5 years. The melanoma patients were grouped into transcriptomic clusters. Cluster A exhibited the highest MSS (80%, 95%CI = 55.1–92.0), significantly better than cluster B (32.9%, 95%CI = 12.0–55.9; *p* = 0.0029). Subcluster B1 (dashed line) had the lowest MSS (16.7%, 95%CI = 1.1–49.3) compared to B2 (short dashes) (50%, 95%CI = 15.2–77.5%; *p* = 0.0014). The survival curves are color-coded for each cluster, with the *p*-values indicating the statistical significance of the survival distributions.

The transcriptomic signatures were also correlated with survival outcomes, with cluster B being associated with poorer MSS compared to cluster A (5-year MSS of 32.9% with a 95%CI = 12.0–55.9; and 80%, 95%CI = 55.1–92.0, respectively; *p* = 0.0029). Within the subclusters B1 and B2, cluster B1 exhibited the lowest MSS (16.7%, 95%CI = 1.1–49.3 and 50%, 95%CI = 15.2–77.5, respectively; *p* = 0.0014) (Figure 2). The median follow-up was 75.4 months (IQR = 45.1) for cluster A, 12.0 months (IQR = 29.4) for cluster B1, and 41.9 months (IQR = 45.3) for cluster B2.

### 3.3. The Transcriptomic Signatures of SNMM and CM Highlight Distinct Profiles and Survival Correlations

**Figure 3:**
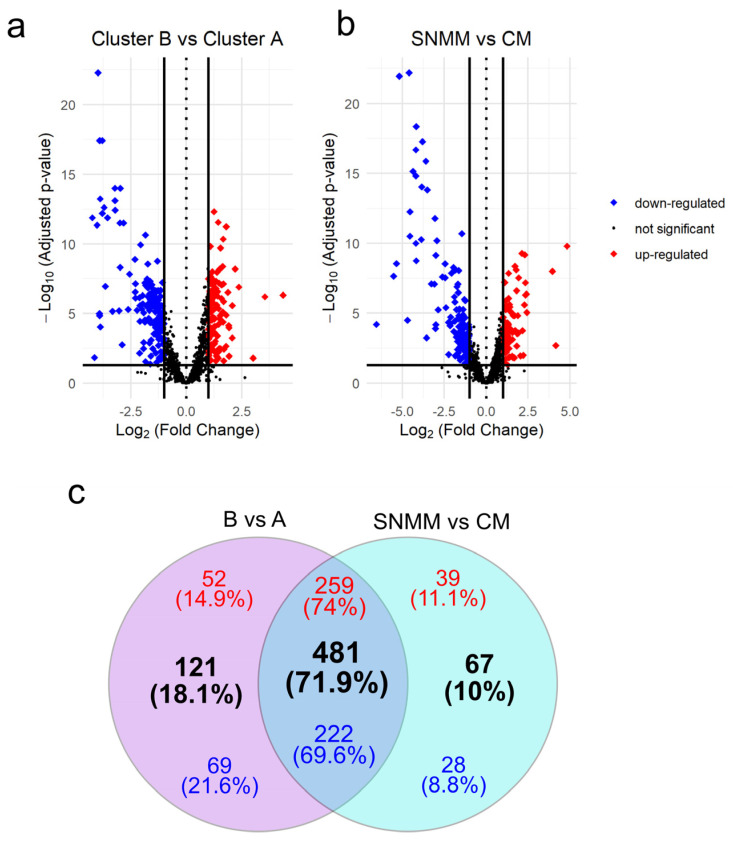
Volcano plots and Venn diagram of differentially expressed genes (DEGs). (**a**) DEGs between cluster B and cluster A; (**b**) DEGs between sinonasal mucosal melanoma (SNMM) and cutaneous melanoma (CM). Genes are marked as upregulated (red), downregulated (blue), or not significant (grey) based on adjusted *p*-values and log_2_ fold change thresholds. Significant genes (*p*-adjusted < 0.05) with a log_2_ fold change ≥1 or ≤−1 are highlighted, with guidelines indicating thresholds for significant expression changes; (**c**) Venn diagram illustrating the overlap and exclusivity of DEGs between the two analyses. Upregulated genes are shown in red, downregulated genes in blue, and the total number of DEGs is indicated in black. Intersections reveal genes that are commonly regulated across both comparisons.

Differential expression analysis comparing the clusters defined by HCA (cluster B vs. cluster A) identified 602 (42.9%) DEGs with an adjusted *p*-value < 0.05, of which 311 DEGs were upregulated and 291 were downregulated (Figure 3a and Supplementary Table S6). A total of 548 (39.1%) DEGs were identified considering the clinical–pathological diagnosis of the tumors (DEGs between SNMMs and CMs), where 298 were upregulated and 250 were downregulated (Figure 3b and Supplementary Table S7). Of these, 481 (71.9%) DEGs, comprising 259 upregulated and 222 downregulated, were common to both approaches (B vs. A and SNMM vs. CM). There were 121 DEGs (18.1%) exclusive to the B vs. A comparison and 67 to the SNMM vs. CM comparison (Figure 3c). Surprisingly, when selecting DEGs resulting from the B vs. A comparisons (Supplementary Figure S2) or DEGs from the SNMM vs. CM (Supplementary Figure S3) as inputs for HCA, tumors were grouped similarly to the initial HCA (clusters A and B; Figure 1), reinforcing the association of molecular signatures with clinical–pathological classifications. To further explore the relationship between the identified genes and patient survival, we performed a log-rank test using the data from all 37 samples to identify genes associated with MSS. This analysis revealed 330 genes with significant *p*-values. Notably, 240 (34.7%) of these genes overlapped with the 602 DEGs identified in the B vs. A comparison, highlighting a strong association between these genes and survival outcomes in melanoma (Supplementary Table S8, Supplementary Figure S4).

Additionally, 359 DEGs were identified between CM from subcluster B2 and cluster A, with 83% of these DEGs also present in the differential expression analysis of B vs. A (Supplementary Table S9, Supplementary Figure S5), further corroborating that certain CMs exhibit more aggressive behavior.

### 3.4. Cell Cycle- and Immune Response-Related Pathways Exhibit Dysregulation in SNMM Compared to CM Tumors

**Figure 4:**
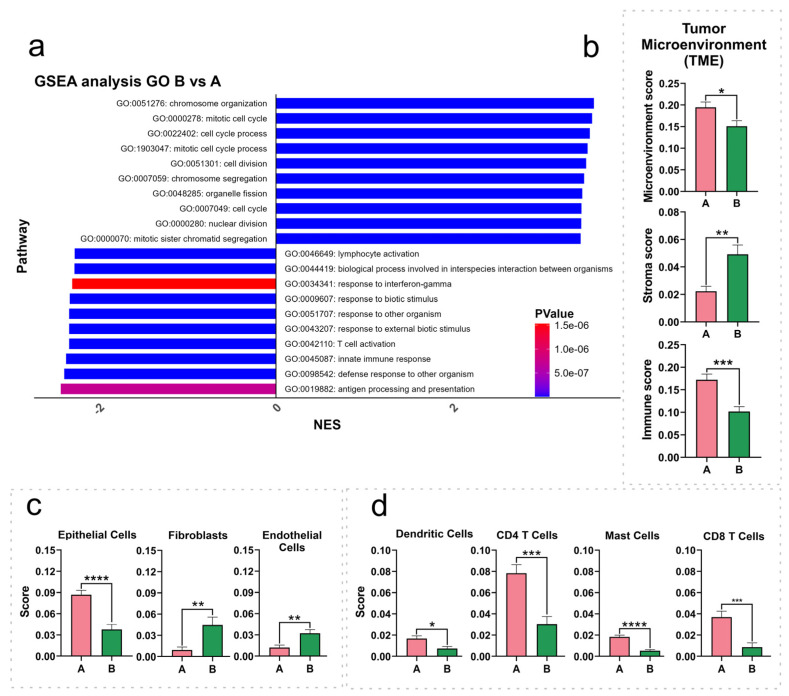
Gene set enrichment analysis (GSEA) and immune population scores across cluster A and cluster B. (**a**) GSEA for cluster B vs. cluster A highlights the top 10 upregulated and downregulated pathways in Gene Ontology (GO) Biological Processes (BP), ranked by normalized enrichment scores (NES), with color-coded *p*-values; (**b**–**d**) relative abundance of immune populations in cluster A and cluster B as determined by the xCell algorithm, categorized into microenvironment, stroma, and immune scores (**b**), specific stroma populations (**c**), and immune populations (**d**). The values displayed in the bar graphs represent the mean, and the error bars correspond to the standard error of the mean (SEM). Differences in significance between the clusters are indicated by asterisks: * (*p* < 0.05), ** (*p* < 0.01), *** (*p* < 0.001), and **** (*p* < 0.0001).

GSEA was conducted on the ranked DEG list (B vs. A), revealing the (GO: BP) positively or negatively enriched (*p* < 0.05) gene sets (Figure 4a). The top 10 negatively enriched (10/10; NES score < −2.27) gene sets (DEGs preferentially downregulated in cluster B, SNMM-enriched) comprised genes linked to immune response processes, including antigen processing and presentation, innate immune response, T cell activation, lymphocyte activation, and response to interferon-gamma. Conversely, the top 10 positively enriched (10/10; NES score > 3.44) gene sets (DEGs preferentially upregulated in cluster B) were related to cell cycle processes, such as chromosome organization, mitotic cell cycle, cell division, and nuclear division. The complete list of all gene sets is available in Supplementary Table S10. Additionally, the results of the GSEA of the DEGs identified in CMs from subcluster B2 vs. cluster A (Supplementary Table S11, Supplementary Figure S6) corroborate these findings and provide insight into why certain CMs exhibit more aggressive behavior.

### 3.5. The Tumor Microenvironment Is Intricately Related to the Clustering Signature

To assess the tumor microenvironment (TME) composition, we utilized the xCell algorithm to estimate the relative abundance of 19 immune and 4 stromal cell types. The immune scores, reflecting the inferred relative abundance and activity of immune cells, were significantly diminished in cluster B (*p* = 0.0002), while stromal scores showed an increase (*p* = 0.0011). The overall TME score, representing the cumulative inferred presence of all relevant immune and stromal cell types, was notably decreased in cluster B (*p* = 0.0185) (Figure 4b).

Upon examining the specific predicted cell populations, we observed reductions in the estimated scores for the epithelial cell (*p* < 0.0001), CD4 T cell (*p* = 0.0003), CD4 memory T cell (*p* = 0.0084), CD4 effector memory T cell (CD4 TEM) (*p* = 0.0105), CD8 T cell (*p* = 0.0006), CD8 central memory T cell (CD8 TCM) (*p* = 0.0079), CD8 effector memory T cell (CD8 TEM) (*p* = 0.0401), dendritic cell (DCs) (*p* = 0.0129), and mast cell (*p* < 0.0001) signatures, in contrast to an increase in the estimated scores for fibroblasts (*p* = 0.0054) and endothelial cells (*p* = 0.0040) in cluster B (Figure 4c,d). The table containing all identified cell signatures and statistics is available in Supplementary Table S12.

**Figure 5:**
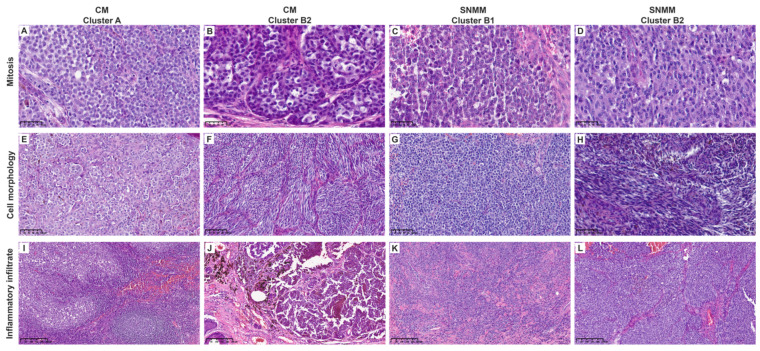
Representative histological images of cutaneous (CM) and sinonasal mucosal melanomas (SNMMs). (**A**–**D**) Mitotic activity (40×). Cluster A (**A**) was similar to clusters B2-CM (**B**) and B2-SNMM (**D**), showing few mitoses per field. Cluster B1 (**C**) exhibited higher mitotic activity. (**E**–**H**) Cell morphology (20×). Cluster A (**E**) predominantly displayed epithelioid morphology, with abundant cytoplasm, less pronounced nuclear pleomorphism, and inconspicuous nucleoli. Cluster B2 (**F**,**H**) also showed abundant cytoplasm but included spindle-shaped cells without evident nuclear pleomorphism or nucleoli. Cluster B1 (**G**) exhibited plasmacytoid morphology with scant cytoplasm, nuclear pleomorphism, and prominent nucleoli. (**I**–**L**) Inflammatory infiltrate (10×). Cluster A (**I**) showed a robust inflammatory infiltrate with lymphoid aggregates. Clusters B2 (**J**,**L**) and B1 (**K**) were similar, displaying minimal peritumoral inflammatory cells. Scale bars are displayed in the bottom left corner of each image.

Blinded histopathological analyses were conducted on all tumors to corroborate the tumor microenvironment (TME) signature data. Cluster B showed a significantly higher mitotic index (median 8, IQR = 8.5) than cluster A (median 3.5, IQR = 6, *p* = 0.0478). Lymphocytic inflammation was predominantly mild (64.7%) in cluster B compared to that in cluster A with moderate–severe inflammation in 65% of the cases (*p* = 0.007). Cluster B also exhibited a lower percentage of TILs (*p* = 0.046) and showed a trend toward non-brisk classification (*p* = 0.097). Epithelioid morphology was predominant in cluster A (80%) compared to cluster B, with 64.7% of microcytic/plasmacytoid morphology (*p* < 0.001), as shown in Table 1. The histopathologic features analyzed for each case are listed in Supplementary Table S13. Representative histopathological images are shown in Figure 5 and Supplementary Figures S7 and S8.

## 4. Discussion

Despite significant advances in the molecular characterization and treatment of melanomas over the last decade, leading to substantial improvements in the overall survival (OS) rates of many patients, SNMM remains a challenging exception. Characterized by its aggressive nature, high metastatic potential, and poor outcomes, SNMM differentiates itself from CM with a distinct molecular profile yet lacks specific therapeutic protocols [18]. This disparity underscores the critical need for targeted research and clinical strategies tailored to SNMM’s unique molecular characteristics to improve prognostic outcomes and address the persistently low OS rates associated with this melanoma subtype. The present study has identified distinct transcriptomic signatures and altered molecular pathways that may elucidate the heightened aggressiveness and worse prognosis of SNMM compared to CM. Specifically, it has identified 602 DEGs related to immuno-oncology and assessed their impact on cell cycle- and immune-related molecular pathways. This altered scenario is associated with a specific TME, characterized by diminished immune cell infiltration and increased cellular components that contribute to angiogenesis and tumoral mitogenic signaling. These insights could enhance our understanding of the distinct nature and treatment responses (e.g., immunotherapies) of SNMM in contrast to CM, offering a more nuanced perspective for clinical oncological research and practice.

The positive enrichment of cell cycle pathways in SNMM-enriched cluster B suggests an increased proliferative capacity, consistent with the observed increase in the mitotic index, a direct measure of cell proliferation and a characteristic of aggressive tumor behavior. Among the pathways positively enriched, several identified DEGs play a crucial role in cell cycle regulation. Genes such as CDC45 and TOP2A, crucial for DNA replication and cell cycle progression, are significantly overexpressed in the SNMM-enriched cluster B. Such overexpression has been linked to reduced OS and MSS in melanoma patients [19,20], with TOP2A overexpression also noted in patients with metastatic melanoma [21]. The upregulation of TEX14 in SNMM may affect the degradation of the REST tumor suppressor, a protein associated with adverse outcomes in cancers such as triple-negative breast cancer, potentially driving the aggressive phenotype of SNMM through disrupted cell cycle regulation [22]. Moreover, genes critical for mitotic spindle assembly, chromosome segregation, and cell cycle checkpoint, including *KIF14*, *NUF2*, *KIF18B*, *TPX2*, *ESPL1*, *CCNE2*, and *CENPF*, are upregulated in the SNMM-enriched cluster B. Alterations in these genes have been associated with a potential compromise in chromosomal segregation accuracy, contributing to the genomic instability characteristic of aggressive tumor types. Indeed, *TPX2* overexpression has been linked to shorter OS in melanoma patients [23]. An in vitro study demonstrated that silencing *KIF18B* in CM cell lines significantly inhibited cell proliferation, migration, and invasion while enhancing apoptosis [24]. *KIF14*, *NUF2*, and *ESPL1*, have been associated with poorer prognosis in various cancers [25,26,27]. Additionally, increased expression of *CENPF* may lead to the premature exhaustion of CD4+ memory T cells and immunosuppression [28], and has been linked to worse prognosis in melanoma patients [20].

Conversely, we observed a negative enrichment of immune-related pathways in the SNMM-enriched cluster B, marked by the downregulation of pivotal genes, such as *CCL19*, *CCL21*, and *CXCL9*, essential for understanding immune evasion mechanisms. These chemokines are crucial for T-cell recruitment and enhancing immune infiltration at tumor sites. Notably, *CCL19* is upregulated in metastatic lesions of long-surviving patients [29], and *CCL21* is linked to substantial infiltration by various T cells and dendritic cells, correlating with favorable prognoses [30,31,32]. However, metastatic cells may downregulate *CCL21* expression via immune-suppressive factors, impacting immune cell localization and altering immune composition [33,34]. CCL19 and CCL21 also influence lymphocyte migration through high endothelial venules (HEVs), which are critical in tumor immunosurveillance and are linked to the overexpression of lymphoid chemokines and genes related to Th1 and naïve T cells [35,36]. Similarly, *CXCL9* correlates with enhanced immune cell infiltration, leading to improved prognosis and increased treatment responsiveness [37,38,39,40]. Notably, genes associated with immune checkpoints, such as *PDCD1* (PD-1), *CTLA4* (CTLA-4), and *CD274* (PD-L1), were significantly downregulated in SNMM-enriched cluster B (*p* = 0.0239, *p* = 0.0021, and *p* = 0.0048, respectively) compared to cluster A. This reduced expression suggests a tumor microenvironment that is less responsive to immune checkpoint inhibitors in cluster B, consistent with the poor responsiveness of SNMM to immunotherapy [41]. These observations highlight the unique immune-related transcriptional profile of cluster B, marked by the downregulation of genes such as *KRT16*, *S100A8/A9* and *TRIM29*, which are also downregulated in this cluster. *KRT16* is linked to better outcomes in metastatic melanoma [42,43], while low levels of *S100A8/A9* correlate with advanced disease stages [44]. Additionally, *TRIM29* is also reduced in metastatic melanoma [45]. The downregulation of these genes highlights the plasticity of melanoma cells, allowing them to evade immune responses, thus enhancing their survival and progression.

The TME analysis estimated a diminished immune score and an elevated stroma score in cluster B, characterized by inferred reductions in CD4+ and CD8+ T cells, and dendritic cells (DCs), and increases in endothelial cells. These computationally derived results align with the observed transcriptomic profiles, suggesting a more immunosuppressive and angiogenic microenvironment in cluster B. While these findings provide valuable insights, they are based on estimations from transcriptomic data and require validation through experimental methods. The presence of tumor-infiltrating lymphocytes (TILs), particularly CD8+ T cells and B cells, strongly correlates with improved survival and tumor control, underscoring their importance as favorable prognostic factors in melanoma [46,47,48,49,50]. In SNMM patients, high densities of CD8+ T cells and natural killer T cells, noted for their immune killing effects, have been associated with better disease control and no disease progression [51]. A recent systematic review analyzing response to immunotherapy in SNMM reported that the presence of brisk tumor-infiltrating lymphocytes was associated with improved recurrence-free survival and overall survival [12].

Interestingly, HCA identified a subgroup of CMs, subcluster B2, sharing molecular characteristics with SNMMs. These CMs show increased aggressiveness, elevated Breslow thickness and mitotic index, together with alterations in the tumor inflammatory profile, suggesting a propensity for aggressive behavior and poorer immunotherapy responses [16,48,50].

This study has several limitations, including a relatively small cohort size, which is a consequence of the rarity of SNMM, and the use of the HTG EdgeSeq Precision Immuno-Oncology panel. While this panel is robust for profiling relevant oncogenic and immune-related genes, it lacks the comprehensive of whole transcriptome sequencing. Future multi-center studies are warranted to validate these findings in larger cohorts and to incorporate more extensive molecular analyses. 

## 5. Conclusions

In conclusion, this study has identified distinct molecular features, including specific genes and pathways involved in cell cycle progression and immune evasion, in SNMMs and certain aggressive CMs. Additionally, it has suggested an altered tumor microenvironment in SNMMs and aggressive CMs, inferred from computational analyses and highlighting a more immunosuppressive and angiogenic profile. These findings highlight the challenges in managing these malignancies due to their inherent aggressiveness and poor prognosis, while offering potential prognostic markers and new avenues for developing targeted treatment strategies to improve patient survival rates.

## Figures and Tables

**Table 1:** Histopathological characteristics of the cohort.

Histopathological Characteristics	Cluster A n = 20	Cluster B n = 17	*p*-Value
Mitosis (mm^2^) ^a^ mean (SD) ^b^	4.8 (3.9)	7.8 (5.3)	0.046
Nucleus pleomorphism, n (%)			0.298
Mild	10 (50.0)	7 (41.2)	
Moderate	10 (50.0)	8 (47.1)	
Severe	-	2 (11.8)	
Nucleolus, n (%)			0.217
Small/inapparent	12 (60.0)	8 (47.1)	
Present	8 (40.0)	7 (41.2)	
Very prominent	-	2 (11.8)	
Necrosis, n (%)			0.088
Absent	20 (100.0)	14 (82.4)	
Present	-	3 (17.7)	
Cytoplasm, n (%)			0.009
Minimum	-	4 (23.5)	
Moderate	7 (35.0)	8 (47.1)	
Abundant	13 (65.0)	5 (29.4)	
Pigment, n (%)			0.797
Absent	5 (25.0)	5 (29.4)	
Mild	9 (45.0)	5 (29.4)	
Moderate	6 (30.0)	7 (41.2)	
Cell morphology, n (%)			<0.001
Epithelioid	16 (80.0)	4 (23.5)	
Fusiform	1 (5.0)	1 (5.9)	
Microcytic/Plasmacytoid	2 (10.0)	11 (64.7)	
Rhabdoid/Pleomorphic	1 (5.0)	1 (5.9)	
Lymphocytic inflammation, n (%)			
Mild	7 (35.0)	14 (82.4)	0.007
Moderate	8 (40.0)	2 (11.8)	
Severe	5 (25.0)	1 (5.9)	
TILs, n (%)			
0–10%	9 (45.0)	14 (82.4)	0.046
20–40%	8 (40.0)	3 (17.6)	
50–90%	3 (15.0)	0 (0.0)	
TILs (Brisk)			
Brisk	6 (30.0)	1 (5.9)	0.097
Non-brisk	14 (70.0)	16 (94.1)	

^a^ mm^2^, square millimeters; ^b^ SD, standard deviation.

## Data Availability

The authors confirm that the data supporting the findings of this study are available within the article and its Supplementary Materials, including the log_2_ (CPM) values (Supplementary Table S14).

## Supplementary Materials

The following supporting information can be downloaded at https://www.mdpi.com/article/10.3390/cancers16244172/s1, Supplementary Methods: Histopathological analysis; Figure S1: Bootstrap dendrogram of gene expression profiles; Figure S2: Hierarchical clustering analysis (HCA) of differentially expressed genes (DEGs) between cluster B and cluster A; Figure S3: Hierarchical clustering analysis (HCA) of differentially expressed genes (DEGs) between sinonasal mucosal (SNMM) and cutaneous melanoma (CM); Figure S4: Kaplan–Meier survival curves for the top 10 genes with lowest *p*-values from the log-rank analysis and Venn diagram comparing DEGs in the B vs. A analysis with log-rank significant genes; Figure S5: Volcano plot and Venn diagram of differentially expressed genes (DEGs) in cutaneous melanoma (CM) from subcluster B2 vs. cluster A; Figure S6: Gene set enrichment analysis (GSEA) for cutaneous melanomas (CM) within subcluster B2 vs. cluster A; Figure S7: Representative histological images (hematoxylin & eosin staining) of cutaneous melanomas (CMs; left panel) and sinonasal mucosal melanomas (SNMMs; right panel) from clusters A and B1; Figure S8: Representative histological images (hematoxylin & eosin staining) of cutaneous melanomas (CMs; left panel) and sinonasal mucosal melanomas (SNMMs; right panel) from cluster B2; Table S1: Patient, sample, and data characteristics; Table S2: Other somatic mutations; Table S3: Gene nomenclature equivalences and eligibility; Table S4: Characteristics of the sinonasal mucosal melanomas cohort; Table S5: Characteristics of the cutaneous melanomas cohort; Table S6: Differential expression analysis of cluster B vs. cluster A; Table S7: Differential expression analysis of SNMM vs. cluster CM; Table S8: Log rank analysis; Table S9: Differential expression analysis of CM in B2 vs. cluster A; Table S10: Gene set enrichment analysis of cluster B vs. cluster A; Table S11: Gene set enrichment analysis of CM in B2 vs. cluster A; Table S12: Cell signatures and statistics; Table S13: Histopathologic features; Table S14: log_2_ (CPM) values.
