# Supplementary material for: Distinct Transcriptomic and Tumor Microenvironment Profiles in Sinonasal Mucosal Melanoma and Aggressive Cutaneous Melanomas
Source: Cancers (Basel). 2024 Dec 14;16(24):4172. doi: 10.3390/cancers16244172 (PMC11674703; doi:10.3390/cancers16244172)
Supplement: Supplementary file 1 [file cancers-16-04172-s001.zip › Supplementary Materials.pdf]

cancers

MDPI

Research article

Distinct Transcriptomic and Tumor Microenvironment Profiles in Sinonasal Mucosal Melanoma and Aggressive Cutaneous Melanomas

Manuel Molina-García[1,2,3,†], María Jesús Rojas-Lechuga[2,4,†], Teresa Torres Moral[1,3,5,*], Jaume Bagué[1,3], Judit Mateu[1,3], Cristóbal Langdon[6], Joan Lop[7], Vinícius Gonçalves de Souza[8], Llúcia Alós[1,7], Mauricio López-Chacón[1,2,4], Sebastian Podlipnik[1,2,3], Cristina Carrera[1,2,3,5], Josep Malvehy[1,2,3,5], Isam Alobid[1,2,4], Rui Milton Patricio da Silva-Júnior[1,3,‡] and Susana Puig[1,2,3,5,*,‡]

Supplementary Methods: Histopathological Analysis

Histopathological analyses were performed in a blinded manner by two experienced pathologists. Tissue samples from cutaneous melanomas (CM) and sinonasal mucosal melanomas (SNMM) were fixed in 10% neutral buffered formalin, embedded in paraffin, and sectioned at 5 μm thickness. Hematoxylin and eosin (H&E) stained sections were digitized using a NanoZoomer Digital Pathology Image (ndpi) scanner (Hamamatsu Nano Zoomer S60) at a resolution of 440 nm/pixel. The scanned images were evaluated using NDPI.view2 software (version 2.9.29) provided by Hamamatsu Photonics.

Quantitative and qualitative assessments were systematically conducted. Mitoses per mm^2 were quantified in the most mitotically active area using high-power fields (40x magnification). Nuclear pleomorphism was graded as mild, moderate, or severe, based on variations in nuclear size, shape, and chromatin distribution. Nucleoli were categorized as small/inapparent, present, or very prominent. Necrosis was recorded as absent or present. The abundance of cytoplasm was classified as minimal, moderate, or abundant, and pigmentation was scored as absent, mild, or moderate. Cell morphology was described as epithelioid, fusiform, microcytic/plasmacytoid, or rhabdoid/pleomorphic. The degree of lymphocytic inflammation was scored as mild, moderate, or severe.

Tumor-infiltrating lymphocytes (TILs) were evaluated following the guidelines established by the International TILs Working Group. Qualitatively, TILs were classified as brisk if they were present diffusely throughout the tumor or along the tumor margins in a continuous manner, and as non-brisk if they were distributed patchily or confined to isolated regions without continuity. Quantitatively, the proportion of the peritumoral area occupied by TILs was assessed and classified into three ranges: 0%–10%, 20%–40%, and 50%–90%. The peritumoral area was defined as the region immediately adjacent to the invasive tumor front, excluding stromal regions beyond this interface. These evaluations relied on high-resolution digital images analyzed using NDPI.view2 software to ensure accuracy and reproducibility. Discrepancies in scoring between the two pathologists were resolved through consensus discussions.

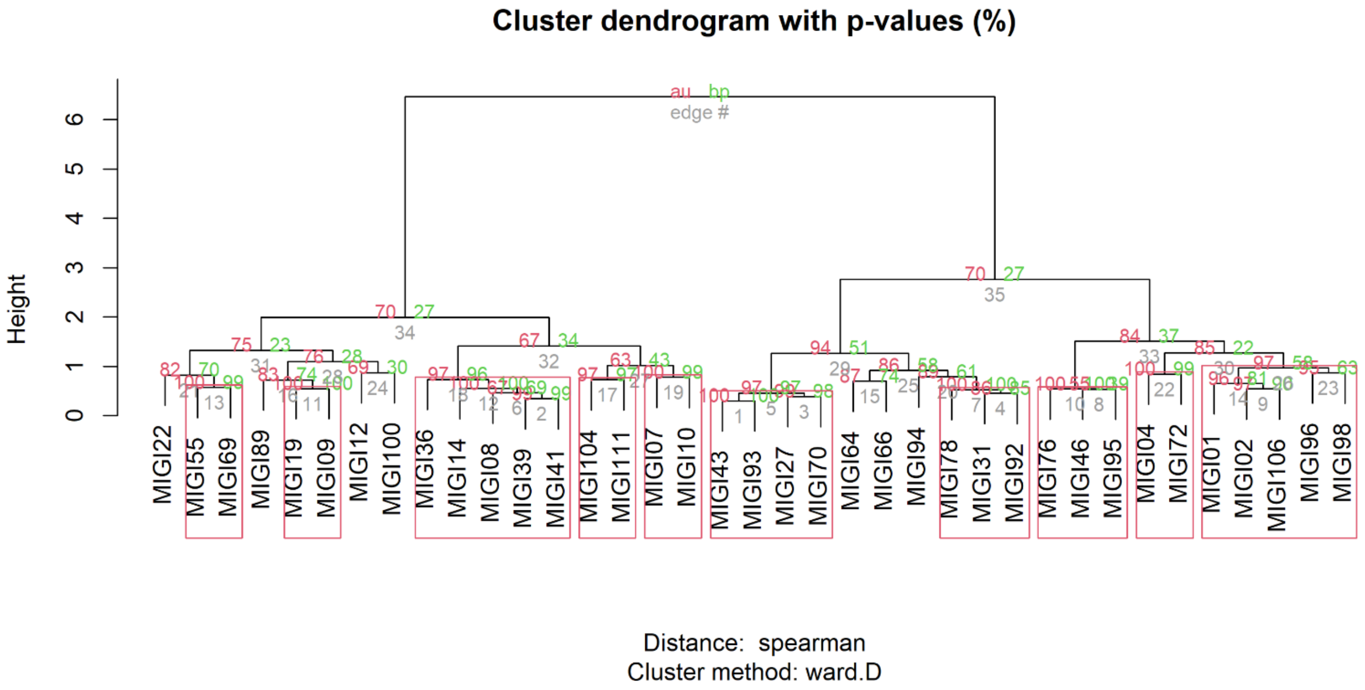

Figure S1. Bootstrap Dendrogram of Gene Expression Profiles. This dendrogram visualizes the hierarchical clustering of gene expression profiles from robust Z-score transformations and Spearman correlation distances using the Ward.D method. Bootstrap values are displayed at the branch nodes: AU (Approximately Unbiased p-value) and BP (Bootstrap Probability), indicating the confidence in the Cluster stability. Shorter edges represent closer genetic similarity between clusters.

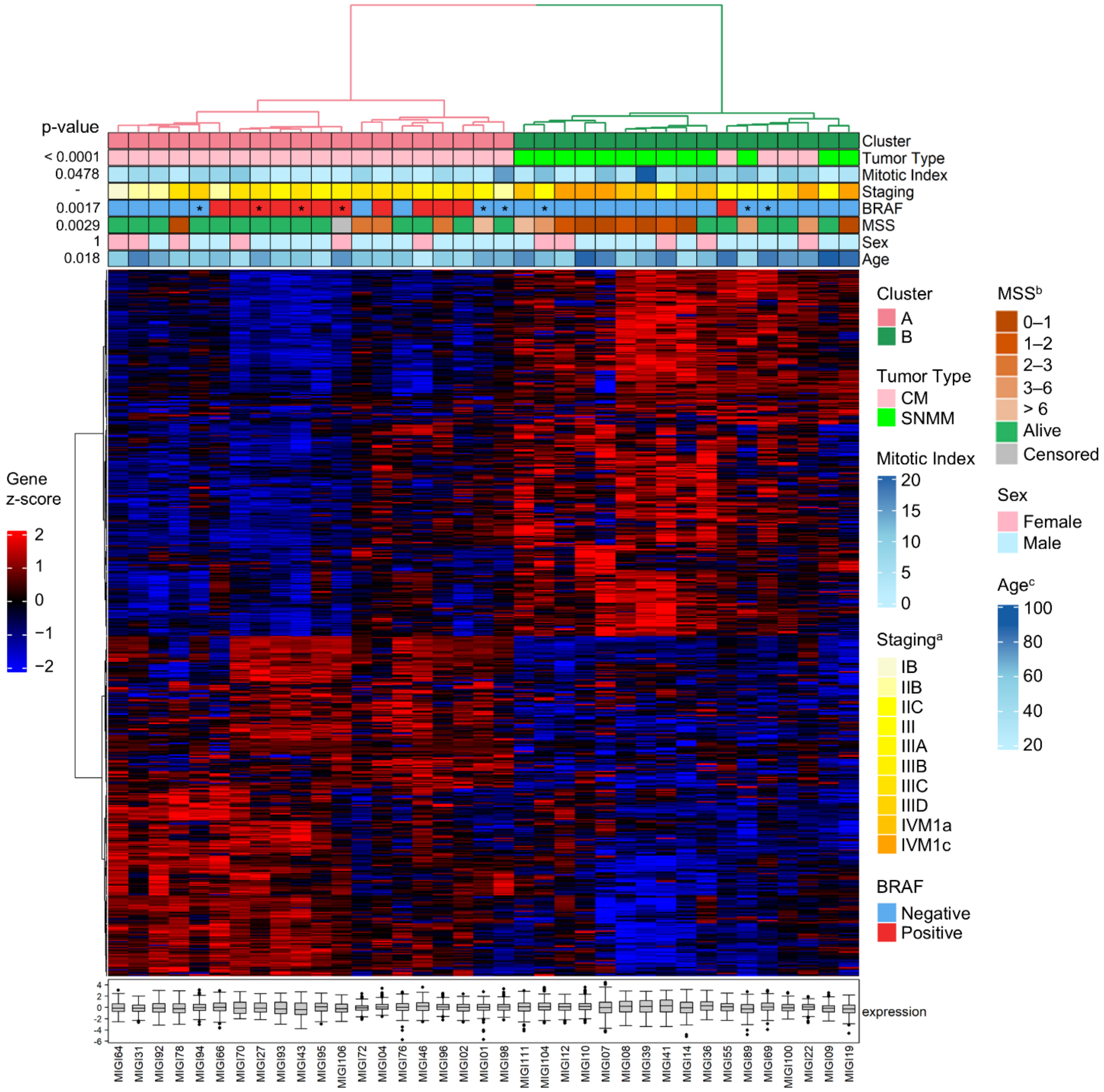

Figure S2. Hierarchical Clustering Analysis (HCA) of Differentially Expressed Genes (DEGs) between Cluster B and Cluster A. The HCA focuses on the expression patterns of (DEGs) between Cluster B and Cluster A. Hierarchical clustering of the samples was performed using Spearman correlation distances and Ward's method. Gene clustering was based on Manhattan distances and Ward.D method. Annotations at the top of the heatmap display metadata with a color code. The expression heatmap reflects gene z-scores, ranging from low (blue: -2) to high (red: 2) expression levels. Boxplots represent the median expression levels of all genes in the panel for each specific sample, showing the interquartile range and outliers to highlight the overall expression variability within each sample. * The BRAF mutation status was obtained from a sample different from the primary tumor. [a] Staging at diagnosis; [b] Melanoma-specific survival (years); [c] Age at diagnosis.

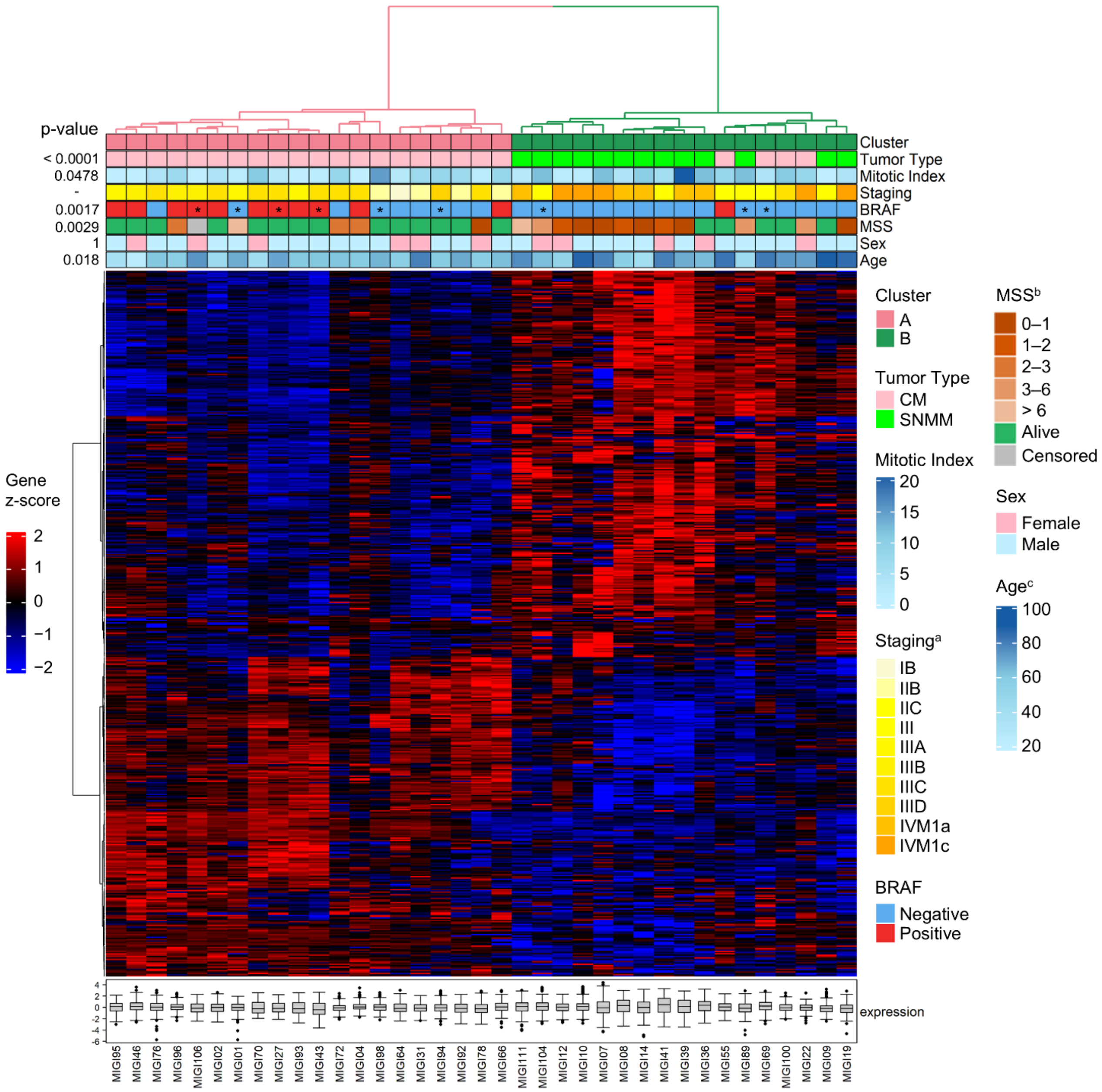

Figure S3. Figure S3. Hierarchical Cluster Analysis (HCA) of Differentially Expressed Genes (DEGs) between sinonasal mucosal (SNMM) and cutaneous melanoma (CM). The HCA focuses on the expression patterns of (DEGs) between SNMM and CM. Hierarchical clustering of the samples was performed using Spearman correlation distances and Ward's method. Gene clustering was based on Manhattan distances and Ward.D method. Annotations at the top of the heatmap display metadata with a color code. The expression heatmap reflects gene z-scores, ranging from low (blue: -2) to high (red: 2) expression levels. Boxplots represent the median expression levels of all genes in the panel for each specific sample, showing the interquartile range and outliers to highlight the overall expression variability within each sample. * The BRAF mutation status was obtained from a sample different from the primary tumor. [a] Staging at diagnosis; [b] Melanoma-specific survival (years); [c] Age at diagnosis.

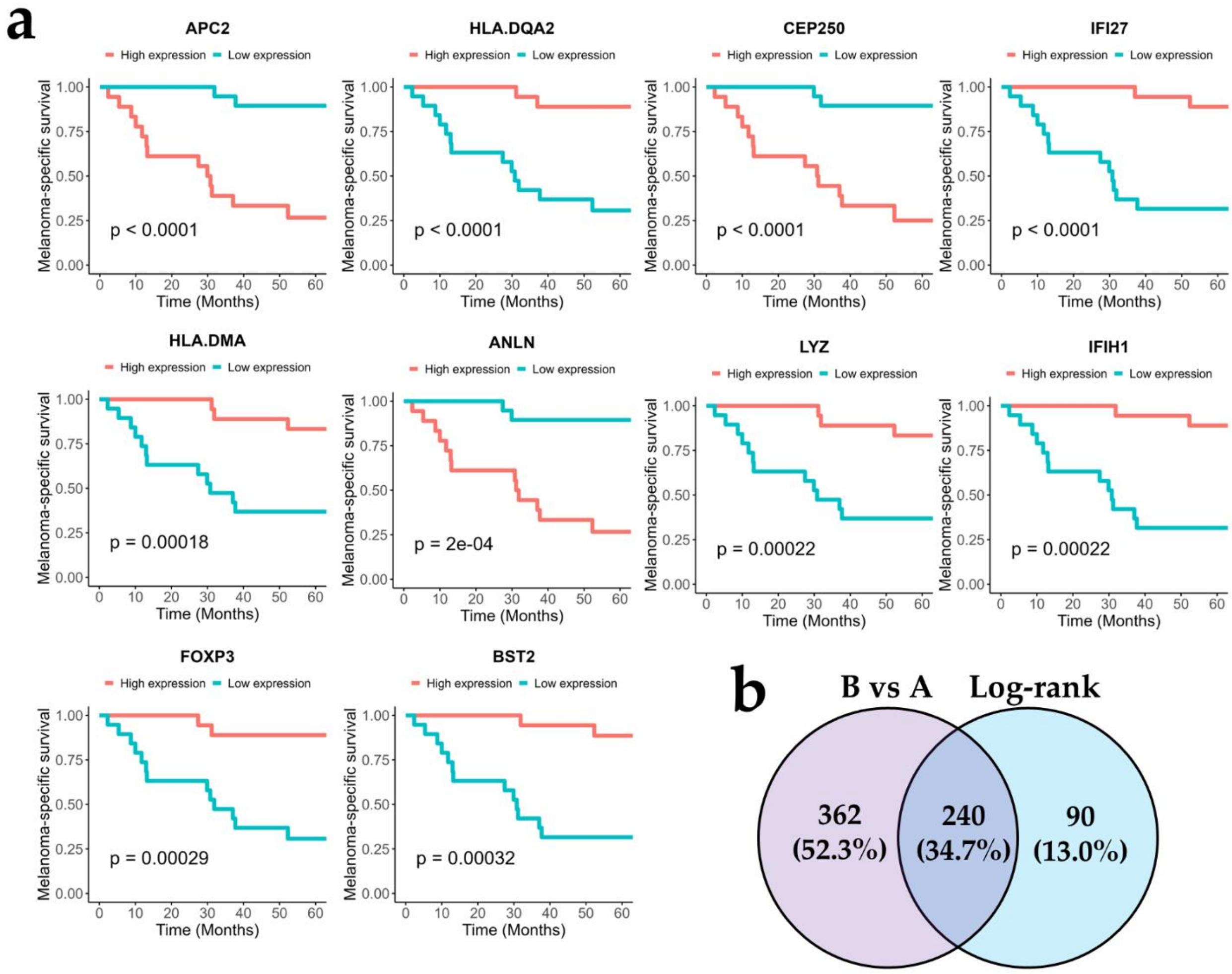

Figure S4. Kaplan-Meier survival curves for the top 10 genes with lowest P-values from the log-rank analysis and Venn diagram comparing DEGs in the B vs. A analysis with log-rank significant genes. (**a**): The Kaplan-Meier curves illustrate melanoma-specific survival (MSS) over time in months, comparing high- and low-expression groups for each of the 10 most significant genes identified by the log-rank analysis. Gene expression levels, measured in counts per million (CPM), were divided based on the median expression value, with "High expression" representing values above the median and "Low expression" representing values less than or equal to the median; (**b**): Venn diagram showing the overlap and exclusivity between DEGs and log-rank significant genes. The intersections indicate genes commonly identified in both analyses.

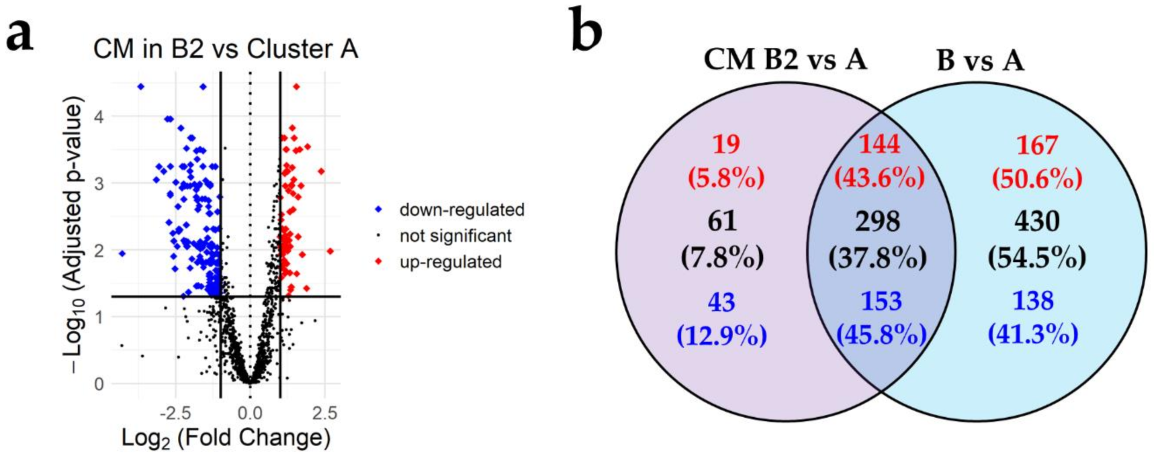

Figure S5. Volcano Plot and Venn Diagram of Differentially Expressed Genes (DEGs) in Cutaneous Melanoma (CM) from Subcluster B2 vs. Cluster A. (**a**): Volcano plot showing DEGs between CM in Subcluster B2 and Cluster A. Genes are categorized as up-regulated (red), down-regulated (blue), or not significant (grey) based on adjusted P-values and log_2 fold change thresholds. Significant genes (P-adjusted < 0.05) with a log_2 fold change ≥ 1 or ≤ -1 are highlighted, with guidelines indicating the thresholds for significance; (**b**): Venn diagram illustrating the overlap and exclusivity of DEGs between the two analyses. Upregulated genes are represented in red, downregulated genes in blue, and the total number of DEGs is indicated in black. Intersections reveal genes that are commonly regulated across both comparisons.

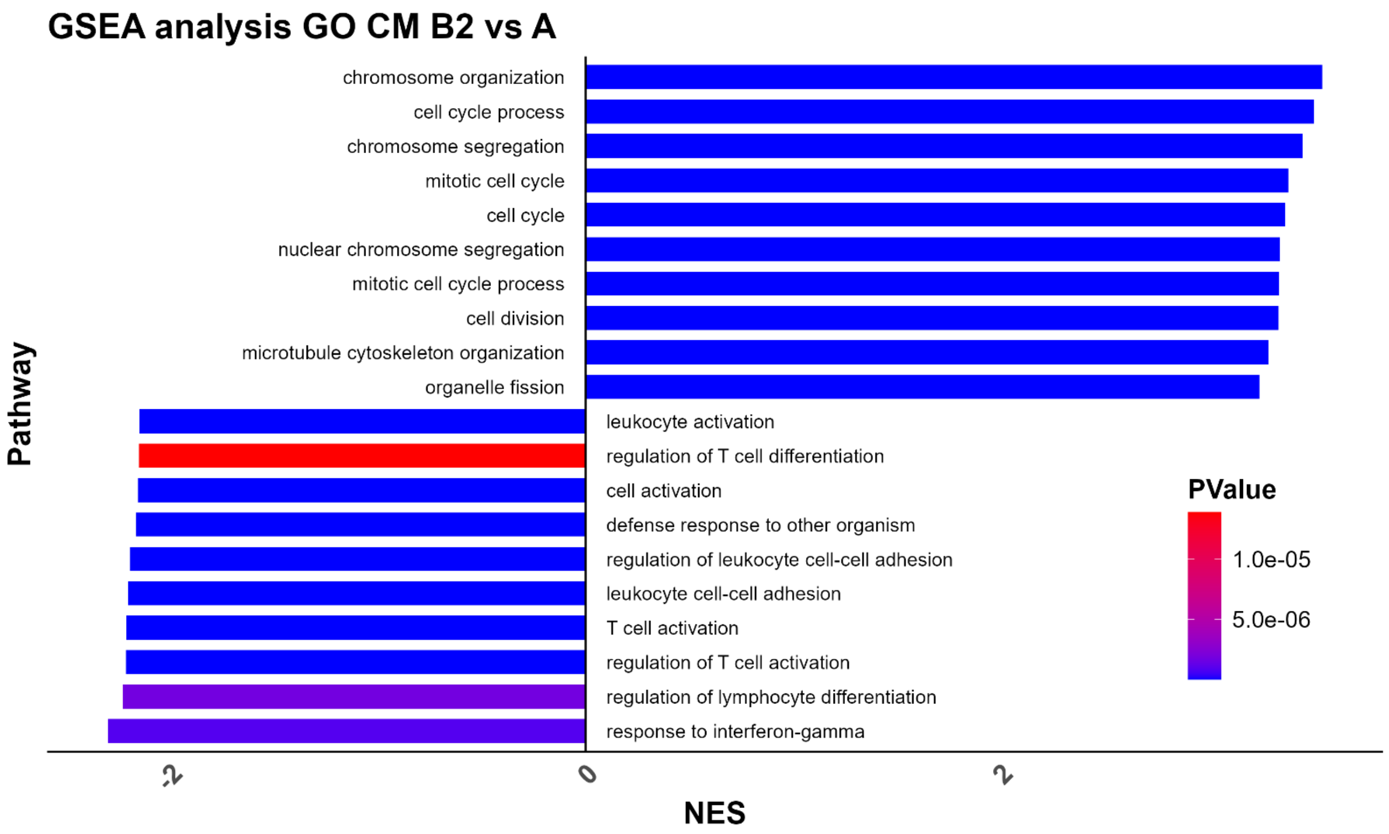

Figure S6. Gene Set Enrichment Analysis (GSEA) for cutaneous melanomas (CM) within Subcluster B2 vs. Cluster A. The GSEA results highlight the top 10 upregulated and downregulated pathways in Gene Ontology (GO) Biological Processes (BP), ranked by normalized enrichment scores (NES). Pathways are color-coded based on their P-values to indicate statistical significance.

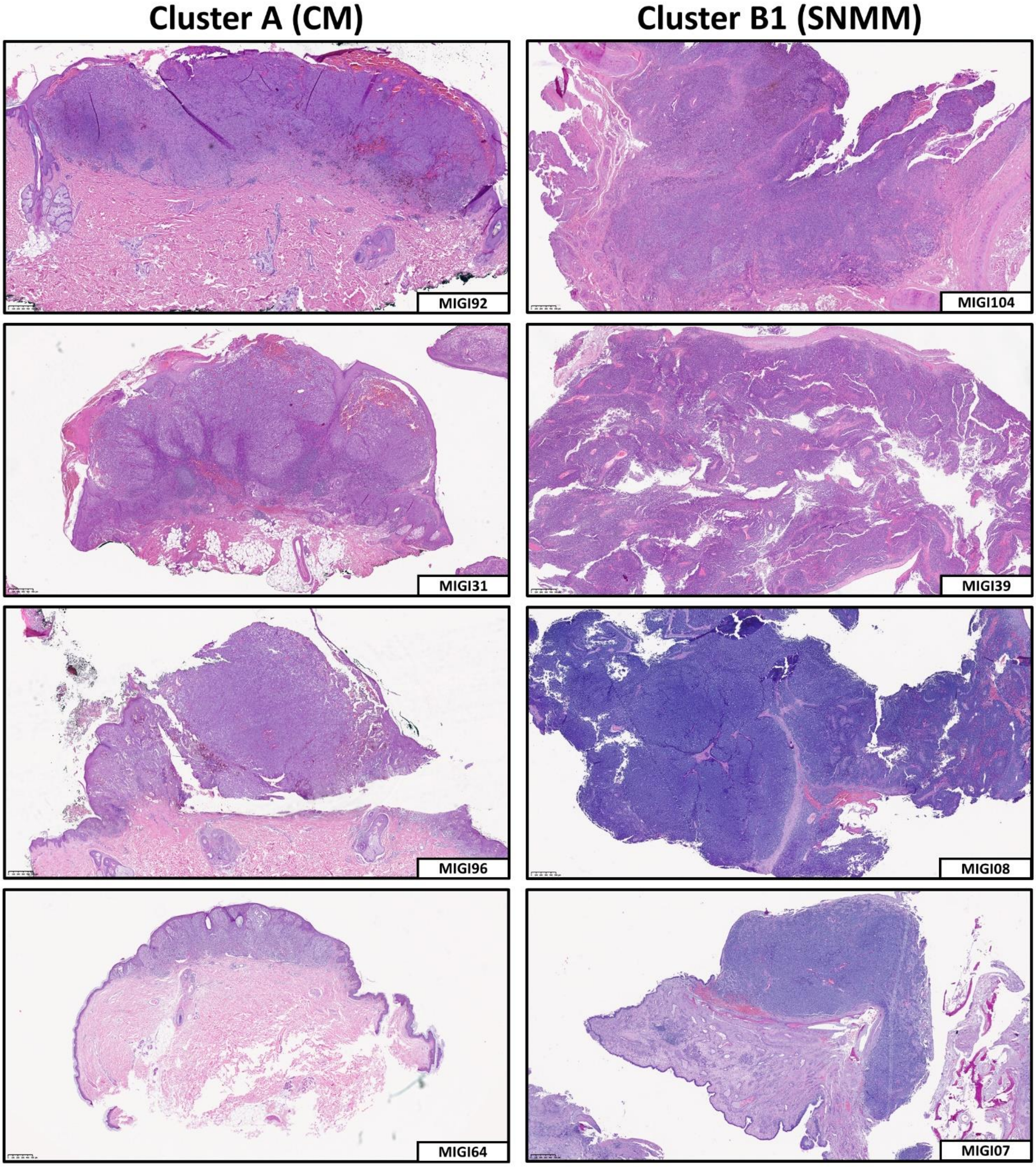

Figure S7. Representative histological images (Hematoxylin & Eosin staining) of cutaneous melanomas (CM; left panel) and sinonasal mucosal melanomas (SNMM; right panel) from clusters A and B1. Low magnification (1.65x), illustrating a panoramic view of the tissue architecture. Scale bars are displayed in the bottom left corner of each image.

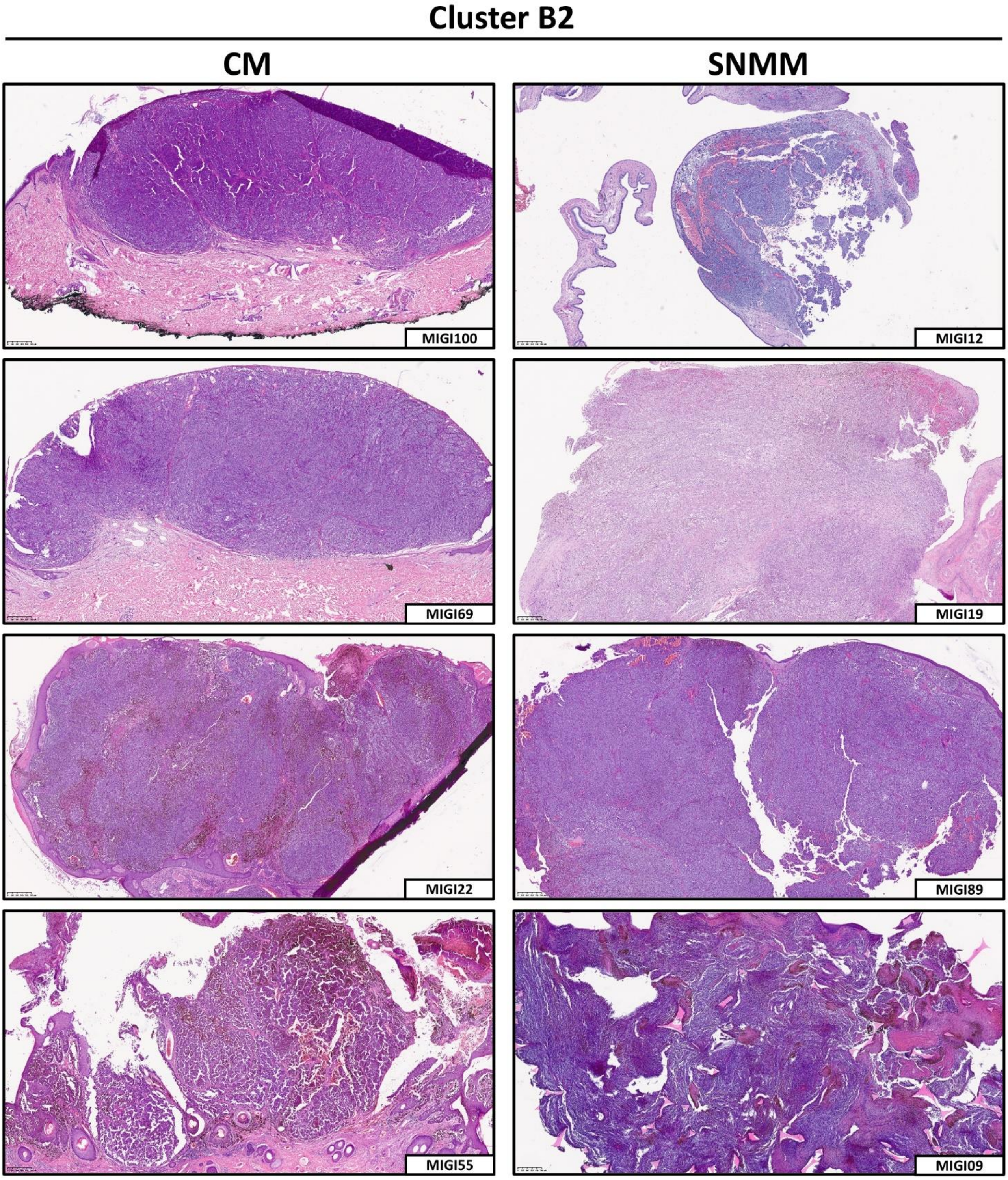

Figure S8. Representative histological images (Hematoxylin & Eosin staining) of cutaneous melanomas (CM; left panel) and sinonasal mucosal melanomas (SNMM; right panel) from cluster B2. Low magnification (1.65x), providing a panoramic view of the tissue architecture. Scale bars are displayed in the bottom left corner of each image.
