# Supplementary material for: Distinct Transcriptomic and Tumor Microenvironment Profiles in Sinonasal Mucosal Melanoma and Aggressive Cutaneous Melanomas
Source: Cancers (Basel). 2024 Dec 14;16(24):4172. doi: 10.3390/cancers16244172 (PMC11674703; doi:10.3390/cancers16244172)
Supplement: Supplementary file 1 [file cancers-16-04172-s001.zip › Supplementary Table S4 and S5.pdf]

Table S4. Characteristics of the Sinonasal Mucosal Melanomas cohort

Characteristics	Sinonasal Mucosal Melanomas n=13
Age (years), mean (SD)[a]	68 (16.3)
Sex, n (%)	
Male	9 (69.2)
Female	4 (30.8)
Tumor location, n (%)	
Nasal cavity	4 (30.8)
Paranasal sinuses	9 (69.2)
Stage[b], n (%)	
III	4 (30.8)
IVa	5 (38.5)
IVb	1 (7.7)
IVc	3 (23.1)
BRAF mutations, n (%)	
Negative	13 (100.0)
Ulceration, n (%)	
Present	12 (92.3)
Absent	1 (7.7)

a: *SD, standard deviation;* ***b***: *Stage using American Joint Committee on Cancer (AJCC) Staging Mucosal Melanoma of the Head and Neck, 8th edition.*

Table S5. Characteristics of the Cutaneous Melanomas cohort

Characteristics		Cutaneous melanomas n=24
Age (years), mean (SD)[a]		61.7 (14.0)
Sex, n (%)		
Male		17 (70.8)
Female		7 (29.2)
Tumor subtype, n (%)		
Superficial spreading		16 (66.7)
Nodular		8 (33.3)
Stage[b], n (%)		
I	IB	2 (8.3)
II	IIB	4 (16.7)
	IIC	2 (8.3)
III	IIIA	3 (12.5)
	IIIB	3 (12.5)
	IIIC	8 (33.3)
	IIID	1 (4.2)
IV	IVM1c	1 (4.2)
Breslow, mean (SD)		3.7 (2.5)
BRAF mutations, n (%)		
Positive		12 (50.0)
Negative		12 (50.0)
Ulceration, n (%)		
Present		13 (54.2)
Absent		11 (45.8)

a*: SD, standard deviation;* ***b****: Stage using American Joint Committee on Cancer (AJCC) Staging Melanoma of the skin, 8*th *edition.*
